# Preliminary revision of the *Physical Education Grit Scale* in Chinese athletes

**DOI:** 10.3389/fpsyg.2023.1136872

**Published:** 2023-03-14

**Authors:** Renfang Zhang, Shenmao Gao, Guangbo Dou

**Affiliations:** College of Kinesiology, Shenyang Sport University, Shenyang, China

**Keywords:** athletes, physical education grit, reliability, confirmatory factor analysis, revision

## Abstract

**Objective:**

The work aimed to revise the *Physical Education Grit Scale* (*PE-Grit*) applicable to Chinese athletes.

**Methods:**

Five hundred and thirty-eight professional athletes from Chinese sports colleges and provincial sports teams were selected by cluster random sampling. Then, the *PE-Grit* was analyzed for project analysis, exploratory factor analysis, confirmatory factor analysis, criterion-related validity analysis, and reliability analysis.

**Results:**

Independent sample *t*-test and item-total correlation analysis of the questions showed that 16 items of the scale had good discrimination. According to the confirmatory factor analysis model, the factor structure consisted of 2 subscales and 4 dimensions (χ^2^/df = 1.827; CFI = 0.961; TLI = 0.953; IFI = 0.961; RMSEA = 0.051). Moreover, Cronbach’s α of the total scale and the 4 dimensions were between 0.751 and 0.865. A significant positive correlation existed between the *PE-Grit*, and self-control, which showed good criterion-related validity.

**Conclusion:**

Revised *PE-Grit* can measure Chinese athletes’ physical education grit for its good reliability and validity.

## 1. Introduction

Grit is important for deliberate practice, motivation, sports performance prediction, and achievements in sports (Fawver et al., 2020; Cormier et al., 2021; Mosewich et al., 2021). Grit, a personality characteristic positively affecting goal achievement and long-term success, reflects passion and persistence for long-term goals. Besides, independent of cognitive ability, it can encourage individuals to work hard and adhere to long-term goals (Duckworth et al., 2007; DiMenichi and Richmond, 2015). Grit includes two aspects: Consistency of interests (CI) reflects the individual’s long-term grit tendency toward goals; persistence of effort (PE) refers to the individual’s tendency to spend more time and energy to achieve long-term goals even when facing setbacks (Duckworth et al., 2007). Students’ motivation, passion, and interest in specific academic backgrounds are related to learning content (Ryan and Deci, 2000; Ulstad et al., 2016). Athletes carry out sports activities in learning and training environments. Physical education, different from other education, is mainly reflected in the physical components of the education process (Guelmami et al., 2022). Therefore, measuring athletes’ grit should focus on the special education background and content. Grit positively affects athletes, while high-level grit is related to psychological factors such as burnout reduction (DeCouto et al., 2019), negative emotions (Doorley, 2021), optimism (Olefir, 2018), perfectionism (Martin, 2018; Fawver et al., 2020), instantaneous positive influence (Rumbold et al., 2022), specific hopes and positive emotions of sports goals (Doorley, 2021), and lower physical anxiety (Auerbach, 2018). Physical education grit can affect athletes’ psychological factors, sports performance, and achievements in specific sports. Therefore, relevant research should be strengthened.

Many tools can measure grit. Grit-O and Grit-S scales developed by Duckworth et al. (2007) and Duckworth and Quinn (2009) are the most widely used and have been revised in many countries. Researchers in the context of pedagogy have developed different measurement tools, such as the *Grit Scale for Children and Adults* (Sturman and Zappala-Piemme, 2017) and *Academic Grit Scale* (Clark and Malecki, 2019). Subsequent research develops the *Grit Psychological Resources Scale* (Schimschal et al., 2022) using the nursing environment, the Triarchic Model of Grit Scale (Datu et al., 2017), and the *Multi-Dimensional Scale of Grit* (Singh and Chukkali, 2021) based on the socialist environment to evaluate grit. Guelmami et al. (2022) develop the *PE-Grit* specifically designed to measure the sports field. The *PE-Grit* is verified in the Arabic version and translated into the English version simultaneously. It consists of *Physical Grit* (*PH*) and *Academic Grit* (*AC*) and has four dimensions: physical interest (PHI), academic interest (ACI), physical effort (PHE), and academic effort (ACE). Grit has a complex structure. The *Grit Scale for Children and Adults* is a single-factor scale that cannot measure grit in sports (Sturman and Zappala-Piemme, 2017). The *Academic Grit Scale*, not including interest and physical input, cannot assess grid in physical education (Guelmami et al., 2022). Compared with the two dimensions of Grit-O and Grit-S, the *PE-Grit* is refined into physical and academic to measure athletes’ grit. Therefore, Guelmami et al. (2022) conducted a reliability and validity test to verify *PE-Grit*. Cronbach’s α of the four sub-dimensions is between 0.83 and 0.86, indicating the good consistency of the scale. KMO = 0.88 in the exploratory factor analysis (EFA). Confirmatory factor analysis (CFA) is used to analyze three different models. Two subscales and four-dimensional third-order factor models, namely Physical Grit (PHI and PHE) and Academic Grit (ACI and ACE), are obtained by calculating the optimal fit index with good construct validity. González-Bernal et al. (2022) verified it in Spanish middle school physical education teaching. Cronbach’s α of four dimensions is 0.81, 0.83, 0.78, and 0.82, which has good construct validity.

Self-control is positively correlated with grit (Toering and Jordet, 2015; Olefir, 2018; Shields et al., 2018; Tedesqui and Young, 2018). Low grit indicates a high anxiety disorder score (Crane et al., 2020). Grit is a personality resource to overcome anxiety in sports if combined with self-efficacy, self-control, and optimism (Olefir, 2018). The *PE-Grit*, developed based on Grit-S, Grit-O, and *Academic Grit Scale*, is assumed to be positively related to Grit-S and self-control.

Currently, researchers do not deeply study grit in physical education in China. Although the adaptability of the *PE-Grit* has been verified in Arabic and Spanish, it has not been revised for Chinese athletes. The work aimed to bring the *PE-Grit scale* from the English context to the Chinese context and the athlete context of sports. Then, the *PE-Grit* for Chinese athletes was developed by verifying its reliability and validity among athletes.

## 2. Objects and methods

### 2.1. Research objects

The work was approved by the Ethics Committee of Shenyang Institute of Physical Education. Combined with online and offline data, 600 professional athletes from Chinese Sports Colleges and Universities and provincial sports teams were selected by cluster random sampling, and 538 samples were finally included. The average age of the valid questionnaires was 21.03 ± 2.56, and the average training period was 5.08 ± 2.32. The samples’ participation in the highest-level competition was as follows: Three people participated at the international level, 80 at the national level, 280 at the provincial level, 154 at the municipal level, and 21 did not participate. Sports included basketball, volleyball, football, badminton, table tennis, tennis, rugby football, skiing, skating, swimming, and track and field. They were divided into two groups for exploratory and confirmatory factor analysis, respectively.

Sample 1: Two hundred and twenty-five samples were randomly selected for exploratory factor analysis, with an average age of 21.01 ± 3.08, including 154 males (68.4%) and 71 females (31.6%). Sample 2: The remaining 313 were used for confirmatory factor analysis, with an average age of 21.04 ± 2.10, including 178 males (56.9%) and 135 females (43.1%).

### 2.2. Research tools

#### 2.2.1. *Physical Education Grit Scale (PE-Grit)*

Guelmami et al. (2022) developed a scale for measuring grit in physical education based on its uniqueness. The scale contains 16 topics and was divided into subscales PH and AC. Besides, it included four dimensions, PHI (e.g., I don’t give many important physics training meetings), ACI (e.g., I am always interested in gaining new theoretical knowledge), PHE (e.g., intense physical exercise will never hinder me), and ACE (e.g., I do not always modify all theoretical topics). The scoring method was Likert’s 7-point scoring, where 1 meant a strong disagreement, and 7 meant a strong agreement.

#### 2.2.2. *Simple Grit Scale* (Grit-S)

The Grit-S on Chinese professional and college athletes was compiled by Duckworth and Quinn (2009) and revised by Liang et al. (2016), including two subscales of interest consistency and persistence effort, and eight items. The scoring method was Likert’s 5-point scoring, from 1 (very inconsistent) to 5 (very consistent), and the four items in persistence effort were scored in reverse. Cronbach’s α of the total scale was 0.843, while that of the two subscales was 0.847 and 0.814, respectively.

#### 2.2.3. *Simple Self-Control Scale* (BSCS)

*A Simple Self-Control Scale* was prepared by Morean et al. (2014) and revised by Luo et al. (2021). Seven items of the scale included two dimensions: Self-Discipline and Impulse Control. The scale adopted Likert’s 5-point scoring from “completely inconsistent” to “completely consistent,” and items 2, 4, 6, and 7 were reverse counting. The higher the score, the higher the level of self-control. Cronbach’s α of the total scale was 0.829, while that of the two subscales was 0.789 and 0.852, respectively.

### 2.3. Research methods

#### 2.3.1. Research procedures

The work has obtained authorization for scale revision from Guelmami. (1) Two English major professors and two sports psychology professors were invited to independently translate the topics of the original scale. The differences in translation were compared to obtain the modified Chinese version. (2) Two more bilingual professors in sports psychology were asked to translate the Chinese version back into English. (3) The similarities and differences between the translated English and the original text were compared. The items with large differences in translation were modified to improve the accuracy of the questionnaire translation. (4) Two sports psychology professors and five psychology graduate students were asked to evaluate the validity of the content so that it conformed to Chinese culture and semantics in terms of expression habits and life customs. For example, academic interest was described as study interest in the work; academic effort was described as an academic investment in the work. (5) Thirty Chinese athletes were randomly selected to complete the scale so that Chinese athletes could easily understand. Then the final questionnaire was formulated.

Questionnaire survey: First, athletes in the test filled out an informed consent. Then, they filled out demographic data and the questionnaire. The testing process was combined online and offline. One hundred and twelve samples were randomly selected to fill out the *PE-Grit* to investigate the reliability of the retest 1 month later.

#### 2.3.2. Statistical methods

Data were analyzed by *SPSS25.0* and *Amos26.0*, while the discrimination was investigated by project analysis. Besides, the concordance coefficient was determined by reliability analysis, while the evidence of structural validity was obtained through confirmatory factor analysis. Meanwhile, the work selected some fitting indices: the chi-square goodness-of-fit statistic, the Tucker-Lewis Index (TLI), the comparative fit index (CFI), the incremental fit index (IFI), and root mean square error of approximation (RMSEA) to determine the model’s fitting degree. Finally, correlation analysis was used to examine the correlation between different variables.

## 3. Results

### 3.1. Project analysis

Two hundred and twenty-five subjects in sample 1 were sorted according to the total scores of PH and AC scales, and the first and last 27% were taken as the high and low groups for independent sample *t*-tests. Some differences in the two subscales’ items between the high and low groups (*P* < 0.001) (see Table 1). Item-total correlation referred to the correlation between the item and the total score of the corresponding subscale. The correlation coefficients of PH and AC items with the total score of the scale were between 0.527–0.735 and 0.545–0.735, respectively. Therefore, the correlation coefficients reached a significant level (*P* < 0.001).

**TABLE 1:** Independent sample *T*-test and item-total correlation coefficient (*n* = 225).

PH			AC		
**Items**	***r***	***t***	**Items**	***r***	***T***
PHI1	0.625**	10.617***	ACI1	0.686**	14.222***
PHI2	0.690**	15.042***	ACI2	0.735**	16.100***
PHI3	0.735**	16.409***	ACI3	0.722**	13.270***
PHI4	0.702**	13.456***	ACI4	0.726**	11.113***
PHE1	0.539**	7.306***	ACE1	0.585**	7.985***
PHE2	0.527**	6.709***	ACE2	0.585**	7.100***
PHE3	0.536**	7.886***	ACE3	0.569**	7.614***
PHE4	0.545**	7.171***	ACE4	0.545**	7.066***

PH, Physical grit subscale; AC, Academic grit subscale; PHI, physical interest; PHE, physical effort; ACI, academic interest; ACE, academic effort.

**P < 0.01; ***P < 0.001.

### 3.2. Validity analysis

#### 3.2.1. Exploratory factor analysis

Exploratory factor analysis of PH was performed with data in sample 1. KMO = 0.766, χ^2^ = 618.246, df = 28, and *P* < 0.001, which were suitable for the analysis. Then, two factors with eigenvalues of 3.057 and 2.023 were extracted by principal component analysis and lithotripsy map test, and the cumulative variance contribution rate was 63.499%. Besides, the factor load of each item after the orthogonal rotation of the maximum variance method was between 0.707 and 0.890 (see Table 2). Exploratory factor analysis of PH was carried out, and the variance weight of factors was 51.854 and 48.146%, respectively.

**TABLE 2:** Results of exploratory factor analysis (*n* = 215).

Items	Mean	SD	Factor loading	Cronbach’s α after deleting items
PHI1	4.453	1.677	0.776	0.839
PHI2	4.293	2.040	0.822	0.840
PHI3	4.507	1.653	0.776	0.834
PHI4	4.578	1.619	0.851	0.834
PHE1	5.467	1.405	0.890	0.840
PHE2	5.022	1.354	0.707	0.838
PHE3	5.244	1.398	0.720	0.837
PHE4	5.133	1.268	0.754	0.837
ACI1	4.556	1.540	0.794	0.845
ACI2	4.502	1.714	0.915	0.845
ACI3	4.396	1.398	0.785	0.849
ACI4	4.631	1.483	0.843	0.845
ACE1	5.253	1.425	0.757	0.846
ACE2	5.204	1.347	0.745	0.838
ACE3	4.867	1.395	0.766	0.840
ACE4	4.729	1.415	0.715	0.842

Similarly, data in sample 1 were used to analyze the exploratory factors of AC. KMO = 0.790, χ^2^ = 682.960, df = 28, and *P* < 0.001, which were suitable for the analysis. Then, two factors with eigenvalues of 3.382 and 1.772 were extracted by principal component analysis and lithotripsy map test, and the cumulative variance contribution rate was 64.424%. Besides, the factor load of each item after the orthogonal rotation of the maximum variance method was between 0.715 and 0.915 (see Table 2). Exploratory factor analysis of AC was carried out, and the variance weight of factors was 54.984 and 45.016%, respectively.

#### 3.2.2. Confirmatory factor analysis

**FIGURE 1:**
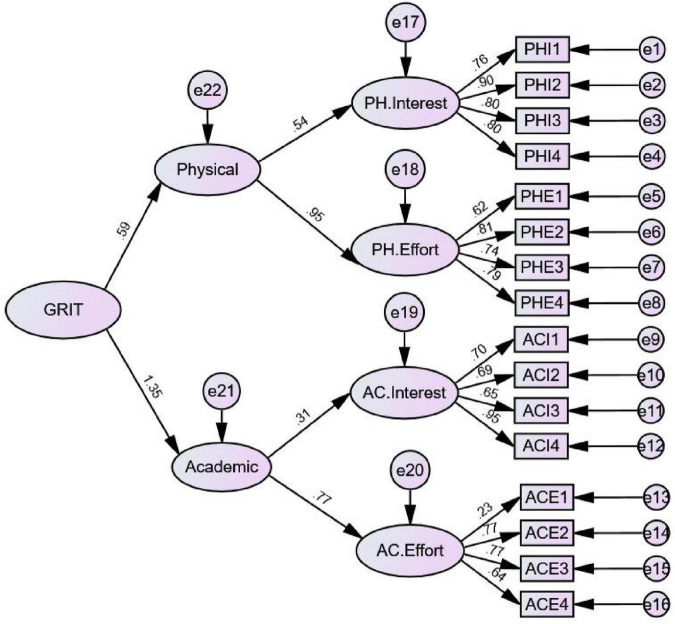
The third-order factor model of the *PE-Grit*. All parameters are standardized and significant at the 0.01 level.

Third-order factor confirmatory factor analysis of data in sample 2 shows that the model fits well (see Figure 1). Table 3 shows fit indices.

**TABLE 3:** Fit indices of confirmatory factor analysis.

χ^2^/df	TLI	CFI	IFI	RMSEA
1.827	0.953	0.961	0.961	0.051

The parameters of the third-order factor model of the *PE-Grit* in Figure 1 are standardized and significant when *P* = 0.01.

#### 3.2.3. Criterion-related validity

The *Grit-S* and *BSCS* were used as criterion questionnaires to test the criterion-related validity of the overall sample. The total score of the *PE-Grit* is positively correlated with that of the *Grit-S* and *BSCS* (see Table 4), indicating that physical education grit is positively correlated with self-control.

**TABLE 4:** Correlation coefficients between the *PE-Grit, Grit-S*, and *BSCS*.

	1	2	3	4	5	6	7	8	9	10	11
1. PE-Grit	1										
2. PH.Interest	0.397**	1									
3. PH.Effort	0.738**	0.397**	1								
4. AC.Interest	0.549**	0.229**	0.105*	1							
5. AC.Effort	0.704**	0.287**	0.535**	0.198**	1						
6. Grit-S	0.311**	0.218**	0.283**	0.186**	0.161**	1					
7. CI	0.838**	0.597**	0.651**	0.449**	0.597**	0.253**	1				
8. PE	0.815**	0.624**	0.621**	0.393**	0.587**	0.236**	0.437**	1			
9. BSCS	0.301**	0.299**	0.157**	0.212**	0.134**	0.385**	0.195**	0.214**	1		
10. Self-Discipline	0.270**	0.302**	0.159**	0.172**	0.074	0.300**	0.180**	0.215**	0.766**	1	
11. Impulse Control	0.231**	0.203**	0.107*	0.176**	0.138**	0.331**	0.146**	0.147**	0.868**	0.346**	1

**P < 0.01.

### 3.3. Reliability analysis

Reliability analysis showed that Cronbach’s α of PH and AC subscales was 0.845 and 0.758, respectively; that of PHI, ACI, PHE, and ACE was 0.865, 0.827, 0.833, and 0.751, respectively; that of the total scale was 0.849. Cronbach’s α after deleting each item was between 0.834 and 0.849, with a maximum of no more than 0.849 (see Table 2). Therefore, there was no need to delete any question items.

Cronbach’s α of PH and AC subscales was 0.889 and 0.863, respectively, after retest reliability; that of PHI, ACI, PHE, and ACE was 0.903, 0.878, 0.888, and 0.858, respectively; that of the total scale was 0.920.

## 4. Discussion

The work revised the *PE-Grit* that was suitable for Chinese athletes. Sixteen items of the questionnaire had good item discrimination after item analysis. Cronbach’s α of PH and AC subscales was 0.845 and 0.758, respectively; Cronbach’s α of PHI, ACI, PHE, and ACE was 0.865, 0.827, 0.833, and 0.751, respectively; Cronbach’s α of the total scale was 0.849. Cronbach’s coefficient α of *PH* and *AC* Subscales was 0.889 and 0.863 after retest reliability, respectively; Cronbach’s α of PHI, ACI, PHE, and ACE was 0.903, 0.878, 0.888, and 0.858, respectively; Cronbach’s α of the total scale was 0.920, which reached the psychometric properties.

Confirmatory factor analysis showed that the internal structure and items’ number of the revised questionnaire were the same as those of the original. The standardized load coefficients of the items on the corresponding subscales were greater than 0.40, with χ^2^/df less than 3, CFI and TFI greater than 0.90, and RMSEA and SRMR less than 0.08. Fit indices met the psychometrics standard, indicating that the scale had a clear structure.

The work adopted the *Grit-S* and *BSCS* as questionnaires to test the correlation validity of the *PE-Grit* criteria, and PHI, ACI, PHE, and ACE *w*ere positively correlated with grit in the *PE-Grit*. Grit is passion and persistence for long-term goals (Duckworth et al., 2007), while grit in sports can promote professional skills’ development by prolonging the time to participate in and adhere to practical activities (Hodges et al., 2017). Physical education grit is the personality embodiment of grit in sports, so they are similar but with differences.

Besides, the work proved that the *PE-Grit* was positively correlated with self-control. Self-control refers to the willpower to suppress impulsive and short-term behavior, while grit refers to the years of effort required to achieve a life goal (Duckworth and Gross, 2014). Self-control is positively correlated with grit (Toering and Jordet, 2015; Olefir, 2018; Shields et al., 2018; Tedesqui and Young, 2018), that is, it is positively correlated with physical education grit. Therefore, the revised athletes’ *PE-Grit* has good reliability and validity and can evaluate Chinese athletes’ physical education grit.

## 5. Limitations and future research directions

The work has some limitations: (1) *PE-Grit* was only tested among Chinese athletes, without considering age groups, so subsequent research should supplement this verification. (2) Gender and sports training background are not controlled, so subsequent research can explore the differences in physical education grit by gender. (3) Since the questionnaire background is a Western culture, subsequent research can develop a scale with Chinese characteristics to measure grit in sports.

## Data availability statement

The raw data supporting the conclusions of this article will be made available by the authors, without undue reservation.

## Ethics statement

The studies involving human participants were reviewed and approved by the Ethics Committee of Shenyang Institute of Physical Education. Written informed consent to participate in this study was provided by the participants’ legal guardian/next of kin. Written informed consent was obtained from the individual(s), and minor(s)’ legal guardian/next of kin, for the publication of any potentially identifiable images or data included in this article.

## Author contributions

RZ reviewed the literature and wrote the manuscript. RZ and SG collected and analyzed data. RZ and GD outlined the structure of the manuscript, reviewed the literature, and wrote the manuscript. All authors contributed to the article and approved the submitted version.
