# Supplementary material for: Preliminary revision of the Physical Education Grit Scale in Chinese athletes
Source: Front Psychol. 2023 Mar 14;14:1136872. doi: 10.3389/fpsyg.2023.1136872 (PMC10043174; doi:10.3389/fpsyg.2023.1136872)
Supplement: Supplementary file 1 [file Table_1.DOCX]

**English version of the PE-Grit.**

Physical interest

- Even if I find physical difficulties during the training session, I find them very important.

- Even when I can do more fun things, I do not miss my physical training.

- I do not give much importance to physical training sessions.

-*I am always interested in new physical exercises in my training sessions.

Physical effort

-Intense physical exercise never discourages me.

- I can maintain adequate physical effort all year round.

- I spare no effort in completing the exercise.

- *During the physical practice, I do whatever is necessary.

Academic interest

- One of my interests is to go deeper into the theoretical side, regardless of the time it takes.

- I am always interested in acquiring new theoretical knowledge.

- Not all theoretical subjects are important.

- *My theoretical duties are very important to me.

Academic effort

- I finish my home exercises, no matter how hard they are.

- I always focus on class to acquire new knowledge.

- I do not always revise all theoretical subjects.

- *I am diligent in all theoretical subjects.

*The item score must be reversed.

**中文版本体育教育毅力量表**

体育兴趣 （1、5、10、13）

-即使我在训练期间发现有一些身体上的困难，也会觉得它们是很重要的。

-即使我有其他更多有趣的事情去做，我也不会错过体育训练。

-*我对体育训练不太重视。

-我在训练期间总是对新的身体练习感兴趣。

体育投入 （3、6、11、14）

-高强度的身体练习从未使我灰心丧气。

-我可以常年坚持足够的体育投入。

-*我不遗余力地投入身体练习。

-在体育锻炼中我会做任何必要的练习。

学业兴趣 （2、8、15、16、）

-我的兴趣之一是不管花费多少时间都要对理论方面进行深入探求。

-我总是对获得新的理论知识感兴趣。

-*不是所有的理论科目都是重要的。

-我的理论学习任务对我来说是非常重要的。

学业投入（4、7、9、12）

-无论有多困难，我都会完成家庭（课外）运动。

-我总是关注课堂上获得的新知识。

-*我不总是温习所有理论科目。

-我在所有理论科目上都很勤奋。

带*项目反向计分
